# Molecular Biology of Meniscal Healing: A Narrative Review

**DOI:** 10.3390/ijms25020768

**Published:** 2024-01-07

**Authors:** Ewa Tramś, Rafał Kamiński

**Affiliations:** Centre of Postgraduate Medical Education, Department of Musculoskeletal Trauma and Orthopaedics, Gruca Orthopaedic and Trauma Teaching Hospital, Konarskiego 13, 05-400 Otwock, Poland; ewa.trams@gmail.com

**Keywords:** meniscus, miRNA, cytokines, angiogenesis, hyaluronic acid

## Abstract

This review provides insights at the molecular level into the current and old methods for treating meniscal injuries. Meniscal injuries have been found to have a substantial impact on the progression of osteoarthritis. In line with the “save the meniscus” approach, meniscectomy is considered a last-resort treatment. Nevertheless, it is important to note that mechanical repair alone may not achieve the complete restoration of the meniscus. A deep understanding of the healing pathways could lead to future improvements in meniscal healing. The inclusion of cytokines and chemokines has the potential to facilitate the process of tear repair or impede the inflammatory catabolic cascade. MicroRNA (miRNA) could serve as a potential biomarker for meniscal degeneration, and RNA injections might promote collagen and growth factor production. The critical aspect of the healing process is angiogenesis within the inner zone of the meniscus. The use of collagen scaffolds and the implantation of autologous meniscus fragments have been successfully integrated into clinical settings. These findings are encouraging and underscore the need for well-designed clinical trials to explore the most effective factors that can enhance the process of meniscal repair.

## 1. Introduction

“Saving the meniscus” is the key to preventing knee osteoarthritis. Meniscus repairs have a higher reoperation rate (20.7%) compared to partial meniscectomy (3.9%), but meniscectomies also have worse long-term outcomes and present increased degenerative changes. Meniscus injuries may initiate osteoarthritis by inducing pathological changes in the subchondral bone and cartilage. A partial or total meniscus removal has been observed to result in an elevation of the peak contact stress within the cartilage and subchondral bone area. Approximately 50–89% of patients with a meniscus injury will develop osteoarthritis (OA) within 20 years [1,2,3,4,5,6].

**Figure 1:**
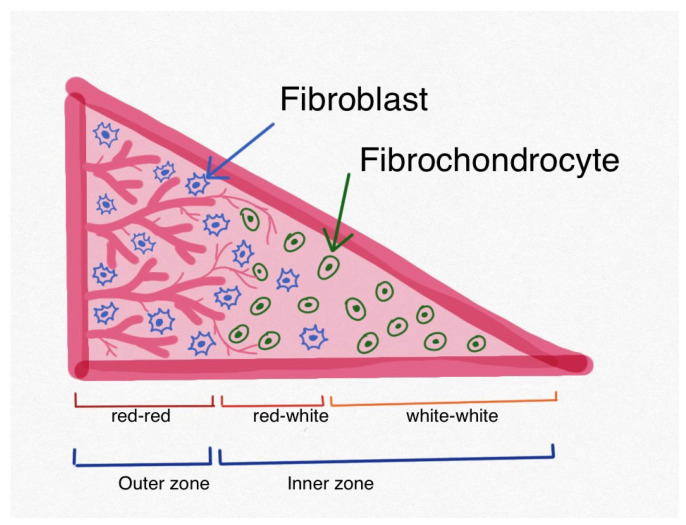
Structure of a meniscus.

The medial (51–74%) and lateral (75–93%) portions of the tibial plateau are covered by the menisci. Their unique semilunar shape facilitates the distribution of loads, joint stabilization, and cartilage protection. They also contribute to proprioception, nutrition, and lubrication [2,7]. The menisci contain water (65–70%), collagen (20–25%), glycosaminoglycans, DNA, adhesion glycoproteins, and elastin. They are divided into three zones, white–white, red–white, and red–red, which represent varying degrees of vascularity. From only 10 to 23% of the peripheral adult meniscus body, known as the red–red zone, receives a blood supply. The other zones are nourished by synovial fluid [7,8]. Each zone has distinct types of cells and molecules. The outer zone, located less than 3 mm from the meniscus rim, constitutes about one-third of the meniscus body. This red–red zone with a good blood supply primarily contains fibroblast cells and forms the extracellular matrix. It is rich in collagen type I, which forms circumferentially oriented fibers in the deepest layer. The mid-body, which is less than 5 mm from the rim, is the red–white zone with residual vascularity. The white–white zone, which is more than 5 mm from the rim, is dominated by fibrochondrocytes. These are anaerobic cells with a few mitochondria that have adapted to survive in a poorly vascularized environment. In this zone, collagen type II predominates, forming radially oriented fibers. The surface of the meniscus is covered by a population of progenitor cells. The inner zone, comprising about two-thirds of the meniscus body, includes both the red–white and white–white zones [7,8,9,10]. The vascularity of the meniscus plays a critical role in the process of healing following an injury. There exist two distinct pathways for meniscal healing, namely the extrinsic and intrinsic ones. Injuries that occur in vascular regions (the outer zone) follow the extrinsic pathway. This process involves the provision of undifferentiated mesenchymal cells by capillaries. Conversely, the self-repair ability of the fibrocartilage and the presence of synovial fluid are essential for the intrinsic pathway, which shows increased responsiveness, particularly in the peripheral zones (inner zone) (Figure 1) [11].

The meniscal tears resulting from axial loading transmitted through the joint or shear forces applied to the meniscus have been classified into various morphological types. These tears could be result of either a chronic or acute injury. In knees with osteoarthritis, the meniscus body is more extruded, and the peripheral external margin has a more convex shape; it bulges more and covers a smaller proportion of the medial tibial plateau compared to healthy knees [12]. In the end stage of OA, the complete maceration of the meniscus body is most frequently observed [13]. The extracellular matrixes in menisci are similar in injured patients and those with osteoarthritis and show the disorganization of collagen fibers. The cells present similar degeneration in both types of menisci: increased chromatin condensation, organelle degeneration, and cytoplasmic vacuolization [14]. Clinically traumatic meniscal tears have better clinical and functional outcomes compared to degenerative meniscus lesions [15].

The current consensus on the treatment of meniscal tears involves the arthroscopic suturing or partial meniscectomy of the damaged area, both of which are considered the gold standard. However, the enhancing of the repair of a meniscal tear located in the avascular zone is crucial. Biological augmentation techniques, such as bone marrow stimulation, fibrin clots, platelet-rich plasma, stem cell therapy, and membrane wrapping, represent the future of meniscus healing when combined with suturing. These methods work at the molecular level to promote regeneration and facilitate vascular ingrowth [8,9]. The studies focusing on the function, regeneration, biology, metabolism, and pathophysiology of the meniscus have enabled us to understand the mechanisms of injury, followed by meniscus repair and healing.

The trials conducted in vivo and in vitro using explant and animal models, and ultimately treating patients in clinical trials, have enabled scientists and clinicians to establish a more effective approach for meniscus healing [16,17].

The aim of this narrative review was to clearly describe the current molecular methods of meniscus repair, summarize the literature, and outline the possible future directions for clinicians.

## 2. Methods

A PubMed search was conducted in October and November 2023, utilizing search terms, such as ‘meniscus’, ‘menisci’, ‘molecular’, ‘TNF-α’, ‘endostatin’, ‘FGF’, ‘biological healing’, ‘interleukin’, ‘cytokine’, ‘Growth Factor’, ‘avascular’, ‘vascularization’, ‘meniscus tear’, ‘meniscus lesion’, ‘hyaluronic acid’, and ‘MiRNA’. The inquiry encompassed articles published over the last 30 years, with a preferential focus on the most recent studies. The objective is to present the latest advancements in meniscus healing research within the genetic domain, reflecting the continuous evolution of this scientific field.

## 3. Biomarkers

The development of osteoarthritis (OA) following a meniscal injury is associated with an increase in inflammatory biomarkers. The cytokines and chemokines in the synovial fluid, which are linked to the absence or presence of meniscal pathology, are well recognized: Interleukin-1β (IL-1β), Tumor necrosis factor-α (TNF-α), Interleukin 6 (IL-6), Interleukin 8 (IL-8), Interleukin 15 (IL-15), Monocyte chemoattractant protein-1 (MCP-1), Macrophage inflammatory protein 1β (MIP-1β), Chemokine ligand 1 (CXCL1), and Interferon γ (IFN-γ) [18]. The presence of joint inflammation and the subsequent release of cytokines and chemokines subsequent to an injury may exert an influence on the meniscal regenerative capacity. The understanding of the roles, routes, and effects associated with meniscus repair has the potential to facilitate the advancement of novel approaches in this domain.

Two strategies exist for molecularly stimulating meniscus healing: either anabolic growth factors are induced to promote healing, or catabolic factors are inhibited to restrict the degradation process. IL-1β and TNF-α expedite the advancement of osteoarthritis through the facilitation of the catabolic cascade. Endostatin demonstrates inhibitory effects on the vascular endothelial growth factor, especially in the avascular zone. Hence, the inhibition of these cytokines may serve as a potential preventive measure against meniscus degeneration subsequent to an injury [19].

### 3.1. Catabolic Growth Factors (GF)

Cell migration to the site of injury is responsible for the healing process. IL-1β and TNF-α have been shown to incite the progression of osteoarthritis by promoting the catabolic cascade. Inflammatory cytokines may also alter the migration of fibrochondrocytes. IL-1β and TNF-α have been found to inhibit the migration of meniscal fibrochondrocytes (MFCs) for 3 days. However, after 7 days, the MFCs return to the baseline levels. Lemmon et al. demonstrated that the addition of an interleukin-1 receptor antagonist (Il-1Ra) to cells previously exposed to IL-1β can restore MFC activity to the control levels. This study confirms that the inflammation of the knee joint negatively influences meniscus healing and affects its repair potential [20].

Cuellar et al., in their prospective study, showed that increased levels of inflammatory cytokines, such as IFN-γ, IL-6, MCP1, and MIP-1β, correlate with meniscal tears in the patients presenting pain symptoms. However, these were almost absent in the asymptomatic patients [21].

Endostatin and chondromodulin-1 (ChM-1) play an anti-angiogenic role and preserve the avascularity of cartilage. Fujii et al. hypothesized that the inner meniscus, by expressing ChM-1, may maintain its avascular feature. They obtained intact lateral menisci during total knee arthroplasty on six patients. In an ex vivo study, ChM-1 was detected mainly in the intercellular region of the inner and superficial zones via immunohistochemical analysis. The endostatin levels were similar in both the inner and outer regions. Western blot analysis detected ChM-1 in the inner region, while endostatin was found in both the zones. These findings suggest that ChM-1 inhibits endothelial cell proliferation and contributes to the avascularity of the inner meniscus region [22].

### 3.2. Anabolic Growth Factors

The application of chemotactic factors at or near a meniscus injury site could enhance the attraction of cells that mediate repair. Fibroblast Growth Factor (FGF) has been found to induce the synthesis of aggrecan and collagen type II, while also promoting the proliferation of chondrocytes, mesenchymal stem cells, osteoblasts, and adipocytes [23,24]. Transforming Growth Factor Beta (TGF-β) stimulates the production of collagen and proteoglycans, and enhances the connections between collagen fibers in the meniscus [25,26]. Bone Morphogenic Growth Factor (BMGF) is known to induce the development of mesenchymal stem cells, exhibit hemostatic properties, and impede the process of matrix disintegration [27]. Insulin-like growth factor (IGF) is widely recognized as a primary mediator for promoting cartilage anabolism, as it enhances cell proliferation and mediates cellular responses [23,28]. The importance of Platelet-Derived Growth Factor-AB (PDGF) in promoting cell proliferation and extracellular matrix formation has been well documented in the literature [29]. Additionally, PDGF has been found to play a significant role in angiogenesis and cellular development.

The study conducted by Lee et al. examined the efficacy of PDGF-coated decellularized meniscus scaffolds in promoting the healing process of the avascular meniscal zone using a bovine model. Decellularized meniscus scaffolds (DMS) were conjugated with heparin to immobilize PDGF-BB. The scaffold was utilized for the seeding of human avascular meniscus cells. An explant model was surgically inserted into a bovine meniscus tear, and then sutured. The control groups, consisting of suture alone, heparin-conjugated DMS, or PDGF-B-coated DMS, did not exhibit any statistically significant variations in cell migration as compared to that of the DMS conjugated with heparin and PDGF-BB. However, the latter resulted in the migration of meniscus cells towards the defect zone. New matrix formation was observed, bridging the space between the native meniscus and the scaffold [30].

Chen et al. assessed the biocompatibility of a self-designed, thermosensitive, injectable hydrogel, which is based on the in situ crosslinking of glycol chitosan (GC) and a multialdehyde-functionalized four-arm polyethylene glycol (four-arm PEG-CHO). The GC/four-arm PEG-CHO hydrogel was utilized in a rabbit model to examine the potential of including Transforming Growth Factor Beta 1 (TGF-β1) in order to promote the process of meniscal repair. The objective was to evaluate if the addition of TGF-β1 may facilitate the differentiation of fibrochondrocyte cells from bone mesenchymal stromal cells (BMSCs). Three groups were studied: control, hydrogel, and hydrogel + TGF-β1. The findings of the study indicate that the hydrogel is a viable scaffold alternative for meniscus tissue engineering. The expression level of the collagen type I and II genes was significantly higher in the TGF-β1 group. The TGF-β1 group exhibited a statistically significant increase in the expression levels of the collagen type I and II genes. Furthermore, it was observed that the group treated with TGF-β1 had the most pronounced protein production of Collagen I, Collagen II, Sox-9, and aggrecan [31].

Cui et al. conducted the transtibial pull-out (TP) repair of the torn anterior horn of a rabbit meniscus. This repair was augmented by the application of PRG, a platelet-rich plasma gel, or with a Platelet-rich plasma (PRP) activator. Three distinct groups were created: the control, transtibial pull-out repair, and experimental groups—TP with PRG. During the surgical procedure, PRG was administered into the transtibial tunnel. Evaluations were conducted after 8 and 12 weeks. Histologically, the PRG group exhibited more collagen fibers between the meniscus and bone compared to the TP group. Additionally, the healing performance was fastest in the PRG group. However, both the TP and TP+PRG groups had a decreased number of inflammatory cells and increased collagen fibers compared to the control. The PDGF and TGF-β1 concentrations in PRG increased 2.46 times and 4.15 times more than those in PRP (*p* < 0.01) [32].

Lu et al. harvested menisci from four patients during total knee arthroplasty (TKA). The menisci were then classified into two distinct categories—native and injured. The tissues from meniscal samples from the first group were prepared. The second group was also divided into two subgroups after creating 5 mm long tears in the inner and outer part of the menisci. Polymerase chain reaction (PCR) analysis showed no VEGF and Hypoxia-inducible factor 1 (HIF-1α) in the freshly isolated meniscal tissues. Cytokines were observed in the cultured menisci, with a notable disparity in VEGF mRNA expression favoring the outer region. The presence of an injury resulted in elevated levels of cytokines, with the most significant increase detected in the outer region. The angiogenic potential of VEGF and HIF-1α suggests that they may have a significant impact on the process of meniscus repair [33].

Nishida et al. investigated the effects of intra-articular injections of Stromal-cell-derived factor 1α (SDF1) on cell migration-dependent rat meniscus regeneration. They administered injections twice at the time of meniscectomy and compared the experimental group with a saline injection group. After one week, a significant increase in the number of CD68-positive and CD163-positive cells was observed in the treatment group. The accumulation CD90-positive cells and CD105-positive cells suggests that meniscus healing is a macrophage-dependent process. After six weeks, a significant increase in the size of the regenerated tissue was observed, as indicated by the Pauli macroscopic score and the meniscus coverage ratio [34].

According to the findings of Mull et al., there is a correlation between the levels of hepatocyte growth factor (HGF) and matrix metalloproteinase 2 (MMP-2) in the synovial fluid and the process of meniscal healing. The study included 48 patients who underwent arthroscopic meniscus repair. After a follow-up of approximately 32 ± 18 months, the patients were categorized into two groups: those with successful surgery, and those with failed surgery (characterized by pain or mechanical complications). Ten of the forty-eight patients required revision surgery. The findings of the study suggest that an increased concentration of hepatocyte growth factor (HGF) and an elevated concentration of matrix metalloproteinase-2 (MMP-2) could potentially serve as indicators of poor meniscal repair [35].

Liu et al. compared patients with isolated unilateral meniscus injuries to a control group. They measured twenty-one biomarkers in their serum and synovial fluid. The results indicated that the PGE2 concentration and MMP activity level were significantly higher in the synovial fluid of the meniscus tear group compared to those of the control. The serum concentration of MMP-10 and IL-6 significantly decreased with time from the injury in the meniscus group. They also compared complex medial meniscus tears with horizontal, bucket-handle, and complex lateral tears. The complex tears had significantly higher MMP-10 levels in the synovial fluid and significantly reduced levels of IL-8 and TNF-α in the sera [36].

Tarafader et al. examined the adverse effects of lubricin (PRG4) on torn menisci and the advantageous influence of genipin on the mechanical properties of hydrogel materials. The findings from the in vitro models demonstrated that lubricin exhibited a preventive effect on both cell and protein adhesion. The researchers proposed that lubricin found in synovial fluid envelopes the injured area and may obstruct tissue integration and healing. To tackle this issue, they modified a bioactive fibrin-based adhesive to attach lubricin to the damaged surface via heparin conjugation. Notably, genipin, when used as a crosslinking agent for fibrin, markedly reduced the rate of fibrin degradation and improved the mechanical properties of the fibrin adhesive. Furthermore, they discovered that heparin-conjugated fibrinogen could modify the healing process of menisci previously coated with lubricin [37].

Goshima et al. investigated the role of basic FGF in meniscus regeneration. They collected synovial mesenchymal stem cells (SMSCs) from patients with osteoarthritis and implanted them in rats that underwent hemi-meniscectomy with or without bFGF. The rats treated with bFGF exhibited a larger regenerated meniscus area, better mechanical properties of the meniscus, and superior histological scores. They found that the addition of bFGF to SMSCs promoted CXCL6 expression, enhancing cell migration, proliferation, and differentiation [38].

## 4. miRNA

MicroRNA (miRNA) is a class of noncoding RNA molecules that play a crucial role in the regulation of gene expression, the suppression of translation, deadenylation, and the post-transcriptional destruction of messenger RNA. Released from cells as apoptotic or necrotic products, established genetic markers like miR-140, miR-27b, miR-16, miR-22, and miR-146a have been detected in OA. miRNA, which is present in the synovial fluid of people with osteoarthritis (OA), is derived not only from cartilage, but also from the synovium and menisci. This suggests that miRNA has the potential to serve as a noninvasive biomarker for monitoring the progression of OA [39,40]. This raises the question: could miRNA be a marker for the progression of meniscus degeneration, or even more importantly, could it influence meniscus healing?

The study conducted by Long et al. investigated the pathological changes in torn menisci with or without a concurrent anterior cruciate ligament (ACL) injury compared to those in healthy menisci. Six menisci for the control group were harvested from the amputees’ legs, and 14 meniscal tissues were collected after partial meniscectomy for both the experimental groups, including radial, flap, and complex meniscal tears. All the meniscal tissues were from the inner zone. They used real-time polymerase chain (rtPCR) reaction along with immunohistochemistry and in situ hybridization to examine the levels of aggrecan gene (ACAN), Disintegrin and metalloproteinase with thrombospondin motifs 5 (ADAMTS5), callagen type 10 alpha 1 chain (COL10A1), CEBPβ, Metalloproteinase 13 (MMP13), and miR-381-3p, miR-455-3p, miR-193b-3p, miR-92a-3p. The findings of the study indicate that the menisci, especially those that are linked to a torn ACL, have a tendency to experience molecular deterioration following an injury. Macroscopically, the torn menisci exhibited more degenerative changes compared to those of the normal menisci. The patients with ACL injuries scored lower on the Lysholm and IKDC assessments than those with an isolated meniscal injury. Interestingly, it was observed that, at the molecular level, only the expression of the ACAN was found to be more down-regulated in the experimental group compared to that of the control group. Furthermore, it was shown that both the miRNA and gene expressions exhibited a general up-regulation in the ACL group, except for ACAN [41].

Kawanishi et al. investigated the influence of miRNA on meniscus healing. They created full-thickness, longitudinal tears on rat medial menisci in the avascular zone (white–white) followed by intra-articular injections of 30 µL of dsRNA. After four weeks, the medial menisci were harvested for histological and immunofluorescence analyses. They demonstrated that the intra-articular injection of miRNA-210 promoted the production of collagen type II alpha 1 chain (COL2A1), VEGF, and FGF2, and the expression levels of these were significantly higher in the experimental group (*p* < 0.05). They reached the conclusion that miRNA-210 has an impact on the healing process of the meniscus, specifically in the white–white zone. In the control group, half of menisci showed no signs of healing, and the rest was only partially healed. In the experimental group, 50% showed complete meniscal healing, and the remaining 50% partially healed, with a significantly higher histological score (*p* < 0.05) compared to that of the control group [42].

Xiao et al. obtained synovial tissues from 12 patients with ACL tears and/or meniscus injuries. The synovial tissues were divided into two groups according to macroscopic and histological criteria: inflammation or control. They performed protein–protein interaction analyses to investigate the differentially expressed RNA and detected 211 differentially expressed miRNAs, 2793 mRNAs, and 3392 long noncoding RNAs (lncRNAs) in the inflammation group, which were compared to the control. They also constructed a competing endogenous RNA network of lncRNA-miRNA-mRNA. These results may contribute to the development of a deeper understanding of post-traumatic osteoarthritis [43].

The concentration- and time-dependent characteristics of cellular and extracellular miRNA in the meniscus, synovitis, and chondrocytes induced by inflammatory cytokines were investigated by Genemaras et al. Six cadaveric porcine knees were employed, with the meniscus, articular cartilage, and synovial membrane sliced into 1 mm^3^ fragments and cultured in normal media. The cells were subsequently stimulated with IL-1β or TNF-α for 8, 16, or 24 h to determine the time-dependent injuries. For the concentration-dependent injuries, the cells were stimulated for 8 h with either 10 or 20 ng/mL of IL-1β or 50 or 100 ng/mL of TNF-α. MiR-146a was significantly up-regulated in all three cell types across every time point and concentration for both the stimuli. In contrast, miR-27b was significantly down-regulated. Mir-16 expression was significantly down-regulated in response to TNF, but not in response to IL-1. MiR-40 was down-regulated only after 24 h in the chondrocytes and meniscus, but not in the synovium. There was no significant difference in any extracellular miRNA ratio for the meniscus cells at 8, 16, or 24 h. In conclusion, miRNA analysis is a potential marker for early OA detection [39].

## 5. Hyaluronic Acid

Hyaluronic Acid (HA), a macromolecular polymer and a component of the extracellular matrix, plays pivotal roles in hemostasis, neovascularization, cell migration, and differentiation. The efficacy of HA in the treatment of osteoarthritis (OA) has been well documented. Injections of Hyaluronic Acid are known to alleviate pain, enhance people’s range of motion, normalize the synovial fluid, and inhibit cartilage degradation. HA exerts its biological activity by binding to various receptors. The interaction between HA-CD44 contributes to both physiological and pathophysiological processes in angiogenesis and inflammation, primarily through the up-regulation of IL-1 receptors. Furthermore, the HA-CD168 axis has a significant role in modulating inflammatory responses and facilitating repair processes [44,45].

Meniscus lesions are intimately associated with the onset and progression of knee osteoarthritis (OA). Acute meniscal tears are a critical factor in initiating cartilage degeneration. In contrast, degenerative meniscus lesions exacerbate the risk of OA by causing hoop stress and diminishing the meniscus’s protective functions, as evidenced in these references [46,47,48]. Viewing the knee joint as an integrated system, where each component interacts molecularly, hyaluronan (HA) may play a vital role in the healing of meniscal tissues. This perspective gains importance in light of the clinical trial outcomes that advocate conservative treatment as the primary approach for degenerative meniscus conditions, positioning meniscus lesions as significant risk factors for OA development [49]. We may hypothesize that a chronic meniscal lesion constitutes a critical phase in OA progression.

Sonoda et al. conducted a study examining the impact of hyaluronan on 35 white rabbits with meniscal injuries in both the peripheral and inner regions. Following meniscal injury and repair, these rabbits were administered intra-articular injections of hyaluronan starting one-week post-surgery and continuing weekly for five weeks. The effects of the hyaluronan injections were contrasted with those of phosphate-buffered saline (PBS). The study found no significant differences in gross morphology, histology, biomechanics, or vascular healing rates between the hyaluronan and PBS groups. However, a noteworthy distinction was observed in the collagen remodeling process, especially in the peripheral region of the meniscus. In the group treated with hyaluronan, there was a significantly higher ratio of reducible collagen crosslinks, suggesting that hyaluronan had a distinct impact on the collagen remodeling in meniscal tissues [50].

Murakami et al. harvested intact lateral menisci during TKA. The meniscus cells were then isolated without collagenase and prepared from 4 mm fragments from both the inner and outer regions, cultured for 1 month, and then stimulated with HA. Migration and proliferation activities as well as the suppression of PGE2-induced apoptosis and mRNA gene expression were analyzed. The study showed that HA significantly increased meniscus cell migration and proliferation in both the inner and outer regions compared to those of the control, and moreover, its effect is concentration-dependent. HA also significantly decreased the number of apoptotic cells by suppressing PGE2-induced apoptosis and caspase3/7 activation. HA increases cell proliferation by up-regulating collagen type 1 alpha 1 chain (COL1A1) and ACAN expression and significantly activating the Phosphoinositide 3-kinase (PI3K) and mitogen-activated protein kinase (MAPK) pathways via the CD44 receptor [51].

Tanaka et al. studied the impact of Hyaluronic Acid (HA) on gene expression in meniscal chondrocytes. They hypothesized that HA’s effects might vary between the inner and outer regions of the meniscus due to differing cell types in these areas. The menisci obtained during total knee arthroplasty (TKA) were processed, stimulated with HA, and cultured. The cells were then subjected to quantitative real-time PCR (qrtPCR) and histological analysis. The study found that HA enhanced the proliferation and migration of meniscal cells in both the inner and outer regions. It increased the expression of COL2A1 in the inner cells and induced the accumulation of collagen type II around the injured side. However, no significant difference in the cell number was observed after the addition of HA in either the superficial layer or the area adjacent to the perforated surface. In conclusion, HA injections could potentially stimulate meniscus healing in the inner region by promoting cell proliferation, migration, and the synthesis of collagen type II [52].

Berton et al. investigated the clinical effect of an HA hydrogel on degenerative meniscus lesions. The study included 40 patients with documented degenerative meniscus lesions and no history of trauma, as confirmed by Magnetic Resonance Imaging (MRI). Each patient received an injection of HA hydrogel. The patients were evaluated using the Western Ontario and McMaster Universities Osteoarthritis Index (WOMAC), Patient’s Global Assessment (PtGA), Clinical Observer Global Assessment (CoGA) at the baseline, 30, and 60 days after injection, and also using the Short Form (36) Health Survey (SF-36) at the baseline and after 60 days. All patients underwent MRI at the baseline and 60 days after treatment. This study showed a significant difference in the patient-related outcome scores after 30 and 60 days compared to those at the baseline. Only one patient needed surgical treatment within a year. The T2 mapping measurements showed healing in 39% of cases in the red and red–white zones of the posterior horn of the medial meniscus, 60% in the white zone of the posterior horn of the medial meniscus, 55% in the red and white zones of the posterior horn of the lateral meniscus, and 65% in the red–white zone of the posterior horn of the lateral meniscus. These promising findings guide future investigations of HA in meniscus healing [53].

Abpeikar et al., in their study, investigated hybrid constructs combining polycaprolactone and a decellularized meniscus extracellular matrix surface modified with HA, gelatin, and selenium nanoparticles in vitro and in vivo in a New Zealand rabbit model. The scaffold promoted the survival and growth of apoptosis-associated speck-like proteins (ASCs) and chondrocytes and influenced meniscus healing by promoting mechanical, functional, and structural functions. In conclusion, this model of tissue engineering yielded good results, and further studies should be considered [54].

## 6. Avascular Meniscal Healing

There has been a higher yearly increase in meniscal repairs compared to that of meniscectomies. Meniscal tears in the outer region, which benefit from better vascularization, typically show positive outcomes post arthroscopy. In contrast, tears in the less-vascularized inner region often result in a lower healing rate, leading to a preference for partial meniscectomy in these cases [11]. As previously mentioned, removing a part of the meniscus can increase joint stress and accelerate osteoarthritis changes. Enhancing healing in the avascular zone could enable the more effective treatment of inner zone tears in the future [49,55].

Deng et al. explored the impact of autologous meniscus fragment implantation on tears in the avascular zone. They divided two groups of New Zealand rabbits into experimental and control cohorts for a study on the lateral meniscus. Both the groups had a full-thickness defect created in the inner two-thirds of the body and anterior horn of the meniscus, which was then covered by an autologous myofascial membrane. In the experimental group, the meniscus was further cut into small pieces (0.125 mm^3^) and placed in the injury site. Meniscus healing was assessed using gross semi-quantitative scoring, microscopic examination with hematoxylin and eosin, and immunohistochemical analysis for proliferating cell nuclear antigen collagen types I and II. The experimental group exhibited a statistically significant improvement in the healing and histological scores over time compared to those of the control (*p* < 0.05). No significant differences in collagen types I and II were observed at the study’s endpoint [56].

Koch et al. conducted a study to assess the efficacy of bone marrow aspirate concentrate (BMAC) in treating avascular meniscus tears. They employed a rabbit model, creating a 4 mm longitudinal tear in the meniscus. In the control group, this tear was treated with a meniscus suture, while the experimental group received a meniscus suture combined with the application of clotted autologous platelet-rich plasma (PRP) or BMAC. The menisci were then harvested for evaluation at 6 and 12 weeks post treatment. Notably, after 12 weeks, while the PRP and control groups exhibited negligible meniscus healing, the BMAC group showed significant healing, with 50% of cases displaying complete recovery and 33% showing partial healing. Histological analysis further revealed that in the PRP group, only 33% exhibited marginal unilateral healing. In contrast, 17% of the BMAC group demonstrated complete integration with the native meniscus, and 50% displayed either complete or partial bilateral integration. These findings suggest that BMAC could be a viable treatment option for such injuries in future clinical applications [57].

In their research, Tarafder et al. explored how different dosages and release rates of connective tissue growth factor (CTGF) and TGF-β3 influence the healing process of meniscus injuries in the avascular zone. They proposed that CTGF initially attracts mesenchymal stem/progenitor cells, followed by TGF-β3 promoting fibrocartilaginous healing. The study utilized bovine menisci with full-thickness cuts in the inner region, treating them with either high (1000 ng/mL) or low (100 ng/mL) concentrations of CTGF and 10 mg of TGF-β3 encapsulated in poly(lactic-co-glycolic acid) (PLGA) microspheres with varying release rates (fast release at a 75:25 ratio of lactic and glycolic acids, and slow release at an 85:15 ratio). Following an 8 week culture period with a monolayer of mesenchymal stem cells (MSCs), the meniscal explants underwent histological, biochemical, and mechanical evaluations. The study concluded that the optimal combination for promoting avascular meniscus healing was 1000 ng/mL of CTGF paired with a slow release (0.29–0.1 ng/day) of TGF-β3 from PLGA microspheres at an 85:15 ratio [58].

Baek et al. conducted a study to stimulate meniscus tissue formation by applying collagen to the avascular region. Medial and lateral human menisci sourced from a tissue bank were segregated into the vascular and avascular regions. Following cultivation, these isolated menisci were then seeded onto an electrospun collagen type I scaffold. In this experiment, a longitudinal tear was created in the avascular zone of a bovine meniscus, where the collagen scaffold was implanted and subsequently cultured for three weeks. The repaired meniscus underwent mechanical, histological, and immunohistochemical evaluations, in addition to magnetic resonance imaging. The results showed that the experimental group exhibited well-integrated neotissue formation within the meniscus. This study underscores the potential of a collagen scaffold, which effectively mimics the natural organization of collagen bundles, as a promising strategy for meniscus regeneration in the avascular zone [59].

## 7. Mechanical Loading and Hypoxia

Mechanical loading on the knee can lead to varying tissue responses. Different molecular and cellular actions occur in response to tissue adaptation. Such loading may cause mechanical stress, leading to injuries to the ligaments, muscles, menisci, and cartilage. However, the specific effects of mechanical loading on meniscal health remain unclear [60,61].

In their study, McNulty et al. determined the effect of mechanical compression on meniscal repair in both inflammatory and normal environments. Medial menisci harvested from pigs were subjected to dynamic, compressive deformational loading applied to 24 cylindrical explant fragments. The samples were loaded for 4 h per day in the presence of either placebo (control group) or 100 pg/mL of IL-1. From the third day onwards, the activities of aggrecanase and matrix metalloproteinase (MMP), along with the release of sulphated glycosaminoglycan (S-GAG) and nitric oxide (NO) production, were measured. IL-1 was found to promote MMP activity in the unloaded samples, but loading inhibited this effect. Furthermore, while IL-1 increased S-GAG release and NO production, loading diminished these releases. Neither IL-1 nor mechanical strain altered aggrecanase activity. The study concluded that dynamic loading might benefit meniscus healing in an inflammatory microenvironment following a meniscus injury, meniscus repair, or osteoarthritis [62].

Irwin et al. investigated the response of meniscal tissue to tensile load under both normal and inflammatory conditions. Porcine medial menisci were harvested from pigs and divided into the inner and outer zones. The samples were then either left unloaded or dynamically loaded with or without the presence of IL-1 (100 pg/mL). RT-PCR was performed to assess the expression of COL1A1, nitric oxide synthase (NOS2), and Transient Receptor Potential Vanilloid 4 (TRPV4). Under neutral conditions, the inner zone showed higher TRPV4, NOS2, and COL1A1 expression levels compared to those of the outer zone. The introduction of IL-1 led to an increase in NOS2 and a decrease in the TRPV4 expression level. Loading reduced TRPV4 expression. Under 5% loading, the inner zone showed the decreased expression of COL1A1. Conversely, the outer zone exhibited increased NOS2 expression under 10% loading. With the addition of IL-1 and 5% loading, the TRPV4 expression level increased and that of NOS2 decreased in both the zones. In the inner zone only, 10% loading increased the expression level of COL1A1 and decreased that of NOS2. This study indicates that mechanical stimulation may be advantageous in treating meniscus injuries by counteracting inflammation-induced damage [63].

Szojka et al. combined mechanical loading with hypoxia. They isolated meniscus fibrochondrocytes, combined them with TGF-β1 and bFGF, and seeded them onto a type I collagen scaffold cultured with TGF-β3 for three weeks. Then, the samples were divided into three groups. The first group underwent 5 days of continuous hypoxia or normoxia (3% or 20% O_2_, after which the tissues were loaded with either dynamic compression or cyclic hydrostatic pressure. The second group experienced 24 h of hypoxia before dynamic compression, with the parts pre-cultured for 3 or 6 weeks and maintained in normoxia for dynamic compression loading. The third group was cultured for 3 weeks in hypoxia with dynamic compression. They found that hypoxia promoted VEGF and SOX9 expression more compared to normoxia, and mechano-hypoxia for 3 weeks improved the mechanical properties and suppressed the hypertrophy markers like COL1A1. These results suggest that under appropriate conditions of loading and hypoxia, the development of meniscus fibrocartilage cells may be supported [64].

Millar et al. harvested the menisci from neonatal pigs and cultured them under hypoxia and normoxia for 14 days. They found that the cells under hypoxia presented preserved round-shaped nuclei and the well-defined deposition of collagen type II, a higher GAG concentration, and an increased expression of collagen type II and SOX-9. This study suggests that hypoxia can enhance meniscal cell maturation [65].

## 8. Discussion

Our study indicates that treating the meniscus at the molecular level may become increasingly important in the future. Given the known impact of a meniscus injury on osteoarthritis (OA) progression, it is crucial to develop new strategies in this field of science. An injured meniscus could even be an initiating factor in OA development, leading to cartilage degeneration and synovial inflammation [1,6]. Olivotto et al. investigated symptomatic meniscal tears. After meniscus repair or meniscectomy, they collected ISAKOS classifications for meniscal tears, macro-scores for synovial inflammation, and Outerbridge classifications for chondral lesions. KOOS scales were also used. They found that suprapatellar synovial hyperplasia and degenerative meniscus changes are predictors of pain and symptoms at the baseline and follow-up and are associated with the severity of chondral damage [66]. Synovial inflammation before meniscus surgery also impacts the patient’s knee pain and symptoms. Meniscal MMP-13 expression is connected with pain as well as with cartilage degeneration [67]. These findings suggest crosstalk between the meniscus, cartilage, bones, and synovial fluid in the injured knee. Several studies confirm this statement. Atik et al. investigated subchondral bone, cartilage, and meniscus samples obtained during total knee arthroplasty with light and transmission electron microscopy. Their study confirmed that all the tissues play roles in OA with crosstalk; however, the factors that initiate the progression of OA remain unknown [6]. Wang et al. screened the differentially expressed genes during OA in the articular cartilage, meniscus, synovium, and subchondral bone. They used four groups of transcription profiles and depicted the potential crosstalk through ligand–receptor pairs. Comparing normal vs. OA samples, they found 626 differentially expressed genes in the articular cartilage, 97 in the meniscus, 1060 in the synovium, and 2330 in the subchondral bone. They constructed a potential ligand–receptor network (55 ligands and 23 receptors in the articular cartilage, 7 ligands and 9 receptors in the meniscus, 64 ligands and 51 receptors in the synovium, and 148 ligands and 152 receptors in the subchondral bones) and identified many potential molecular crosstalk pathways within and between the tissues. They found that TGF-β1, FN1, and TNC might play roles in communication between tissues in OA, and then support OA progression [68].

Therefore, it is important not only to repair damaged tissue, but also prevent the progression of OA. Platelets contain growth factors (PDGF, TGF- β, VEGF, EGF, IGF, and FGF) which are secreted after platelets activation [69]. As mentioned before, these growth factors could play a significant role in meniscus healing. Li et al., in their systematic review and meta-analysis, compared the failure rate and patient-reported outcomes in meniscus repair augmentation with and without PRP. They found that the PRP group had a significantly lower failure rate, as well as statistically significant improvements on the VAS and KOOS scales compared to those of the non-PRP group [70]. Bone marrow aspirate concentrate (BMAC) also contains growth factors, such as PGF, TGF- β, and BMP2-7, which also provide anti-inflammatory and healing effects to the damaged meniscus [71]. Massey et al. treated patients with BMAC after arthroscopic meniscus repair. They found an improvement in pain after three months compared to that of the control group; however, the PROMs, after one year, showed no difference between the groups [72]. Mariani et al. demonstrated an improvement in osteoarthritis symptoms with a single injection of BMAC. The Tegner, VAS and WOMAC scores improved in all the groups according to Kellgren–Laurence (grades 1, 2, and 3) [71]. Fokter et al. investigated the injection of an engineered calcium phosphate compound in a 76-year-old woman. The tissue was harvested after four years during total knee arthroplasty and histologically examined. They found pain relief, but no influence on the natural progression of knee OA [73]. Collagen meniscal implants are another way to replace the meniscus after partial meniscetomy. Lucidi et al. analyzed 156 cases with Collagen Meniscal Implant (CMI Stryker) at a mean of 10 years of follow-up. The overall survival rate was 87,8%, and 85% of patients were satisfied or partially satisfied. The study identified Outerbridge grade 3–4 as a risk factor for failure [74]. Kohli et al. searched databases for studies with CMI, NUsurface (active implants), or Actifit (Orteq LTD) implantation. They found 262 patients with Actifit, 109 patients with CMI, and 65 patients with NUsurface. The failure rates were, respectively, 18%, 6.5%, and 16.9%. The clinical outcomes improved significantly postoperatively [75]. While these studies are quite promising, due to the high failure rate related to cartilage degeneration, we do not recommend using meniscal scaffolds as the sole treatment, but suggest further studies with the addition of biological therapies which can improve the healing rate.

Growth factors are mentioned above in PRP or bone marrow, but a promising new avenue in meniscus diagnostic or potentially healing is miRNA. There are studies that have shown great healing potential in OA with miRNA. Si et al. investigated the injection of miRNA-140 and found that it promotes collagen II expression and inhibits MMP-13 and ADAMTS-5 expression in human OA cartilage-derived chondrocytes and synovial fluid, consequently alleviating OA progression in rats [76]. Tao et al. found that the overexpression of miRNA-183 inhibited the pain-related factors (TRPV1, Nav1.3, Nav1.7, and Nav1.8) and proinflammatory cytokines, like IL-6, IL-1β, and TNF-α, in a mouse model. As a result, it ameliorated pain and could be a possible therapeutic molecule in OA treatment [77]. There is still a lack of studies on this molecule in meniscus samples, but the current studies showed that miRNA could be valuable to researchers for understanding the mechanism of meniscus degeneration in OA and exploring new diagnostic biomarkers for early-stage OA, as well as exploring the relationship between the meniscus and cartilage degeneration. Only one included study showed a possible approach to meniscus treatment after an injury, but this study was performed on rats. Another three studies [39,41,43] diagnosed miRNA as a marker in meniscus degeneration or as an early marker of OA because, as we know, meniscus injury/degeneration is a key factor in faster OA progression. There is a strong need for experiments that use the healing potential of miRNA on the meniscus, as previously proven in OA using a rat model. It is crucial to investigate potential reverse expression of miRNA, which can be involved in meniscus healing. This is because natural inflammatory molecules could negate the potential benefits, especially if the miRNA exhibits pro-inflammatory or pro-apoptotic tendencies. Identifying their inhibitors could be a critical step in meniscus healing. For instance, MiR-16 expression was significantly down-regulated in response to TNF-α. Similarly, MiR-146a was down-regulated by both TNF-α and IL-1 β, and MiR-140 was down-regulated by both the factors, but within a specific time frame (24 h) [39]. On the other hand, targapremir-210, a click chemistry reagent, inhibits pre-MiR-210 [78]. The human leukocyte antigen complex group 11 (HCG11) inhibited the expression of MiR-381-3p by targeting lncRNA [79]. Additionally, the rno-miR-455-3p inhibitor, a chemically modified oligonucleotide, hybridizes with mature miRNA and silences it [80].

## 9. Conclusions

**Figure 2:**
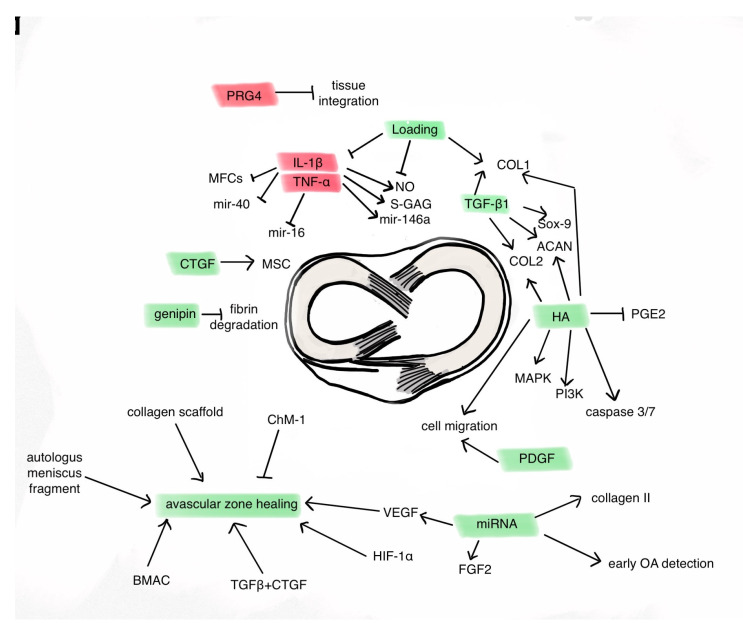
Signaling pathways associated with meniscal healing. (➔ stimulation, ⊣ inhibition).

Our narrative review summarizes meniscus regenerative methods at the molecular level (Table 1). The future of meniscal injury treatment might reside at the molecular level. Degenerative meniscus tears, which often accompany chondral lesions, should be regarded as the key component of knee osteoarthritis. The molecular treatments have shown positive outcomes in chondral degeneration cases (Figure 2).

Promising preclinical studies, both in vitro using explant models and in vivo on animal models, have demonstrated the potential for meniscus healing at the molecular level. However, there is a notable gap in clinical trials from phases I to IV in this area. There is a pressing need for well-designed randomized studies that build on the previous research, focusing particularly on the healing of the avascular meniscus zone and the enhancement of healing post suture with the addition of cytokines, chemokines, or miRNA.

Our two studies (level 1, randomized controlled trials) have demonstrated the efficacy of bucket handle meniscus biological healing through a bone marrow venting procedure (BMVP) and PRP injections. Comparatively, the PRP injections resulted in a significantly higher healing rate (85% vs. 47%) and markedly improved functional outcomes as measured with the IKDC, WOMAC, and KOOS scales [81]. Similarly, the trial utilizing BMVP reported a significantly higher healing rate (100% vs. 76%) with substantial improvements noted in the IKDC, KOOS, WOMAC, and VAS [82]. Additionally, our clinical observations indicate exceptionally positive outcomes with the PRP treatments and the rehabilitation of degenerative meniscus conditions commonly associated with concurrent cartilage deterioration. These outcomes are a result of the molecular crosstalk among all the mentioned knee tissues.

In our view, surgeons should consider strategies to enhance the meniscus’s healing potential beyond merely treating it as damaged tissue for suturing or removal. Various potential treatments, such as PRP, bone marrow aspirate, and possible the application of specific biomarkers inhibitors/enhancers alongside a meniscus suture or scaffold, exist. However, there remains a critical need for more level I clinical trials that investigate the combination of these different methods.

## Figures and Tables

**Table 1:** Meniscus regenerative methods.

	Author	Investigation
Catabolic Growth Factor	Cuellar [21]	INF- γ, IL-6, MCP1, MIP-1 β
	Fujii [22]	Endostatin, ChM-1
Anabolic Growth Factor	Lee [30]	PDGF
	Chen [31]	TGF- β 1
	Cui [32]	PDGF, TGF- β 1
	Lu [33]	VEGF, HIF-1 α
	Nishida [34]	SDF1
	Mull [35]	HGF, MMP-2
	Liu [36]	PGE2, MMP-10
	Tarafader [37]	PRG4
	Goshima [38]	FGF
miRNA	Long [41]	miR-381-3p, miR-455-3p, miR-193b-3p, miR-92a-3p
	Kawanishi [42]	miRNA-210
	Xiao [43]	network of lncRNA-miRNA-mRNA
	Genemaras [39]	miR-146a, miR-27b, miR-16, miR-40
Hyaluronic Acid	Sonoda [50]	HA
	Murakami [51]	HA
	Tanaka [52]	HA
	Berton [53]	HA
	Abpeikar [54]	HA
Avascular healing	Deng [56]	Meniscus small pieces
	Koch [57]	BMAC
	Tarafader [58]	CTGF, TGF- β 3
	Baek [59]	Collagen scaffold
Environmental stimuli	McNulty [62]	Mechanical compression
	Irwin [63]	Dynamic loading, IL-1
	Szojka [64]	Mechanical loading, hypoxia
	Millar [65]	Hypoxia

## Data Availability

Not applicable.
